# PNPLA3 and SERPINA1 Variants Are Associated with Severity of Fatty Liver Disease at First Referral to a Tertiary Center

**DOI:** 10.3390/jpm11030165

**Published:** 2021-03-01

**Authors:** Georg Semmler, Lorenz Balcar, Hannes Oberkofler, Stephan Zandanell, Michael Strasser, David Niederseer, Alexandra Feldman, Felix Stickel, Pavel Strnad, Christian Datz, Bernhard Paulweber, Elmar Aigner

**Affiliations:** 1 First Department of Medicine, Paracelsus Medical University Salzburg, 5020 Salzburg, Austria; georg.semmler@meduniwien.ac.at (G.S.); lorenz.balcar@meduniwien.ac.at (L.B.); s.zandanell@salk.at (S.Z.); m.strasser@salk.at (M.S.); a.feldman@salk.at (A.F.); b.paulweber@salk.at (B.P.); 2 Division of Gastroenterology and Hepatology, Department of Internal Medicine III, Medical University of Vienna, 1090 Vienna, Austria; 3 Department of Internal Medicine, General Hospital Oberndorf, Teaching Hospital of the Paracelsus Medical University Salzburg, 5110 Oberndorf, Austria; c.datz@kh-oberndorf.at; 4 Department of Laboratory Medicine, Paracelsus Medical University Salzburg, 5020 Salzburg, Austria; h.oberkofler@salk.at; 5 Department of Cardiology, University Heart Center Zurich, University Hospital Zurich, University of Zurich, 8006 Zurich, Switzerland; David.Niederseer@usz.ch; 6 Department of Gastroenterology and Hepatology, University Hospital Zurich, University of Zurich, 8006 Zurich, Switzerland; Felix.Stickel@uzh.ch; 7 Medical Clinic III, Gastroenterology, Metabolic Diseases and Intensive Care, University Hospital RWTH Aachen, 52074 Aachen, Germany; pstrnad@ukaachen.de

**Keywords:** PNPLA3, SERPINA1, advanced chronic liver disease, cirrhosis, NAFLD, ALD

## Abstract

Single nucleotide polymorphisms (SNPs), including *PNPLA3 rs738409* and *SERPINA1 rs17580,* have been identified as risk modifiers in the progression fatty liver disease (alcoholic (ALD) or non-alcoholic (NAFLD)). While *PNPLA3* has been studied in various settings, the value of both SNPs has so far not been addressed in a real-world cohort of subjects referred for a diagnostic work-up of liver disease. Thus, liver disease severity was assessed in 1257 consecutive patients with suspected ALD or NAFLD at the time of referral to a tertiary center. Advanced chronic liver disease (ACLD) was present in 309 (24.6%) patients and clinically significant portal hypertension (CSPH) was present in 185 (14.7%) patients. The *PNPLA3* G-allele was independently associated with a higher liver stiffness measurement (LSM; adjusted B: 2.707 (1.435–3.979), *p* < 0.001), and higher odds of ACLD (adjusted odds ratio (aOR): 1.971 (1.448–2.681), *p* < 0.001) and CSPH (aOR: 1.685 (1.180–2.406), *p* = 0.004). While the *SERPINA1* Z-allele was not associated with a higher LSM or the presence of ACLD, it was independently associated with higher odds of CSPH (aOR: 2.122 (1.067–4.218), *p* = 0.032). Associations of the *PNPLA3* G-allele and the *SERPINA1* Z-allele with CSPH were maintained independently of each other. The presence of both risk variants further increased the likelihood of ACLD and CSPH.

## 1. Introduction

Following genome-wide association studies, several single nucleotide polymorphisms (SNPs) have been identified as modifiers in the progression of chronic liver disease (CLD). On the one hand, the *rs738409* G-allele encoding the *I148M* variant of *patatin-like phospholipase domain-containing protein 3* (*PNPLA3*) has been linked with non-alcoholic fatty liver disease (NAFLD) and non-alcoholic steatohepatitis in the general population [1,2]. Moreover, it has been shown to increase progression to cirrhosis in alcoholic liver disease (ALD) [3,4,5,6], the severity of alcoholic steatohepatitis [7], the risk of disease progression in patients with chronic hepatitis C [8] and to augment the risk of developing hepatocellular carcinoma (HCC) [9,10]. On the other hand, the *Serpin Family A Member 1* (SERPINA1 rs17580) Pi*Z-allele has been associated with liver cirrhosis, inflammatory activity and fibrosis stage [11]. Additionally, SERPINA1 Pi*Z has been associated with increased odds of liver-related mortality [12]. Recently, Strnad et al. [13] reported that the SERPINA1 Pi*Z-allele was associated with disease severity and an increased risk of developing cirrhosis in patients with NAFLD and ALD. Surprisingly, this association was independent of *PNPLA3*, *TM6SF2 (Transmembrane 6 superfamily 2 rs58542926*) and *MBOAT7* (*membrane-bound O-acyltransferase domain-containing protein 7 rs641738*), and the association with the development of NAFLD/ALD cirrhosis was stronger than that of other risk variants (including *PNPLA3*) [14]. These findings raise questions about the impact and clinical usefulness of these variants on the severity of liver disease in clinical practice. We aimed to assess these questions in a cohort of consecutive subjects who had been referred to a tertiary referral center for a diagnostic work-up of liver disease.

## 2. Materials and Methods

### 2.1. Patients and Definitions

**Figure 1:**
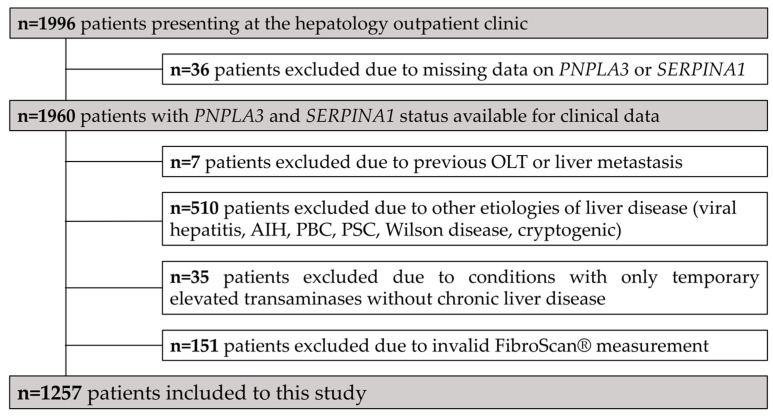
Study flow chart. From the 1996 eligible patients, 739 patients were excluded, and 1257 patients were included in this study. AIH—autoimmune hepatitis; OLT—orthotopic liver transplantation; PBC—primary biliary cirrhosis; PSC—primary sclerosing cholangitis.

All patients who attended the hepatology outpatient clinic of the Paracelsus Medical University Salzburg, for the first time between June 2016 and July 2020, were evaluated for inclusion in this retrospective cross-sectional analysis. Analysis of the *PNPLA3 rs738409* and *SERPINA1 rs17580* risk variants (Pi*MZ, Pi*Z, and Pi*ZZ) commenced during clinical routine on 16 June 2016, as part of the laboratory evaluation of all patients at first time assessment, and the data from 1960 subjects were collected for analysis. Patients were only included if the etiology of liver disease was either suspected to be NAFLD or ALD, and if information on the *PNPLA3 rs738409* and *SERPINA1 rs17580* genotypes was available (*n* = 36 patients were excluded because of missing genotypes; Figure 1). Additionally, patients were excluded if they had previously undergone liver transplantation, or if they had a liver metastasis of an origin other than HCC (*n* = 7), viral hepatitis B or C (*n* = 297), autoimmune liver disease (i.e., autoimmune hepatitis (AIH); *n* = 38), primary biliary or sclerosing cholangitis (PBC/PSC; *n* = 113), Wilson disease (*n* = 5) or an undetermined etiology of liver disease (*n* = 57). Subjects without CLD exhibiting conditions that only caused temporarily elevated transaminases (*n* = 35) and with invalid liver stiffness measurements (LSM; *n* = 151) were also excluded.

The amount of alcohol consumed was estimated as the number of drinks per week, and the average daily alcohol consumption over the week was calculated from that information; significant alcohol consumption was defined as ≥3 drinks per day for men and ≥2 drinks for women [15]. We used this threshold to categorize whether fatty liver disease (FLD) had an alcoholic origin (i.e., ALD.). Hepatic decompensation and decompensated advanced CLD (dACLD) were defined as the presence or a history of ascites, hepatic encephalopathy (HE) or variceal bleeding. ACLD was defined as an LSM ≥10 kPa, unequivocal clinical signs of cirrhosis or a history of hepatic decompensation [16]. Clinically significant portal hypertension (CSPH) was also defined as unequivocal clinical signs of portal hypertension (i.e., varices or portosystemic collaterals) and/or a history of hepatic decompensation and/or an LSM ≥20 kPa.

### 2.2. LSM and Controlled Attenuation Parameter Measurement

The LSM and controlled attenuation parameter (CAP) measurements were performed by transient elastography using a FibroScan^®^ (Echosens, Paris, France). The measurements were carried out after patients fasted for a period of at least 3 h. The M- and XL-probes were chosen as suggested by the manufacturer or based on the expertise of the hepatologist. Patients were instructed to lie in a dorsal position with the right arm in abduction, and measurements were performed on the right lobe of the liver through the intercostal spaces [17]. The reliability of LSM was defined in accordance with previously established criteria [18].

### 2.3. Genotyping for SNPs

Genomic DNA was collected from the peripheral blood samples according to the standard procedures for genotyping *PNPLA3 rs738409* and *SERPINA1 rs17580* [19]. The 5-nuclease allelic discrimination TaqMan genotyping method was performed using pre-designed assays from Applied Biosystems (Foster City, CA, USA), according to the manufacturer’s instructions, on a ViiA7 instrument (Applied Biosystems, Forster City, CA, USA). For quality control, 10% of the samples were genotyped in duplicates. 

### 2.4. Statistical Analyses

Statistical analyses were performed using IBM SPSS Statistics 26 (SPSS Inc., Armonk, NY, USA) and GraphPad Prism 8 (GraphPad Software, La Jolla, CA, USA). Depending on their distribution, continuous variables were reported as mean ± standard deviation (SD) or median (IQR). Categorical variables were presented as numbers and proportions. Comparisons of the continuous variables were performed using the Student’s t-test or Mann–Whitney U test, as applicable. Proportions of patients were compared using the *χ*^2^-squared test. Multivariable linear regression analyses were used to assess the associations between SNPs and LSM. Additionally, multivariable logistic regression analyses were used to determine their association with the presence of ACLD and CSPH. The following covariates were included into regression analyses: age, body mass index (BMI) and alcohol consumption. To assess the influence of risk variants, genotypes were graded binomially for the presence of the *PNPLA3 G*-allele (C/C vs. G/C or G/G) and the *SERPINA1 Z*-allele (M/M or M/S vs. M/Z or Z/Z). To test whether the allele frequency observed in our study conformed to a population in the Hardy–Weinberg equilibrium, we performed *χ*^2^-squared tests as previously described [20], with values of ≥3.84 indicating sample ascertainment bias. A two-sided *p* value ≤ 0.05 was considered statistically significant.

### 2.5. Ethics

This study was approved by the Ethics Committee of Salzburg. As this is a retrospective analysis, the requirement of written informed consent was waived by the ethics committee.

## 3. Results

### 3.1. Study Population and Patient Characteristics

In total, 1996 patients were eligible for inclusion in this study (Figure 1). Following predefined exclusion criteria, 1257 patients with FLD were included in the final analysis. The majority of patients were male (*n* = 759, 60.4%), with a mean age of 52.7 ± 15.2 years (Table 1). NAFLD was the more common etiology (*n* = 1048, 83.4%), while a smaller proportion was classified as ALD (*n* = 209, 16.6%). The mean BMI was 27.2 ± 5.0 kg/m^2^, the median LSM was 5.9 kPa (IQR: 4.5–9.2) and the mean CAP was 277 ± 68 dB/m. In total, 286 (22.8%) and 144 (11.5%) patients had an LSM ≥ 10 kPa and ≥ 20 kPa, respectively. Overall, ACLD was present in 309 (24.6%) patients and CSPH in 185 (14.7%) patients, while 76 (6%) had dACLD.

### 3.2. Prevalence of Risk Alleles

The PNPLA3 wild-type (C/C) was present in 623 patients (49.6%), whereas the heterozygous variant (G/C) and the homozygous mutant-type (G/G) were present in 495 (39.4%) and 139 (11.1%) patients, respectively (Table 1). Thus, the p-allele frequency was 0.6925, the q-allele frequency was 0.3075 and χ^2^ was 7.1308 when compared to an ideal/normal population in the Hardy–Weinberg equilibrium. For SERPINA1, the allele distribution was as follows: 1147 with M/M (91.2%), 43 with M/S (3.4%), 66 with M/Z (5.3%) and 1 with Z/Z (0.1%). Furthermore, the p-allele frequency was 0.9730, the q-allele frequency was 0.0270 and χ^2^ was 0.0074 when compared to an ideal population in the Hardy–Weinberg equilibrium. These results indicate that the PNPLA3 G-allele accumulates in patients referred for a diagnostic work-up of CLD, while we found no such evidence for the SERPINA1 Z-allele.

### 3.3. Differences between PNPLA3 and SERPINA1 Genotype Variants

Patients with the PNPLA3 G-allele had significantly higher Fib-4 scores (*p* = 0.036), and aspartate aminotransferase (AST; *p* < 0.001) and alanine transferase (ALT; *p* = 0.001) levels compared with patients without this risk allele. Patients with the SERPINA1 Z-allele were primarily female (56.7% female vs. 38.7% male; *p* = 0.003) and showed higher cholinesterase (*p* = 0.003) levels (Supplementary Table S1), whereas all other laboratory parameters and disease severity scores were similar between carriers and non-carriers of the risk variants. Next, we compared LSM and CAP values among carriers of the respective risk alleles. Differences were only observed for LSM between the PNPLA3 genotypes (GC/GG: 6.1 (4.6–11.2) vs. 5.8 (4.5–8.0); *p* = 0.002), and for CAP between the SERPINA1 genotypes (MM/MS: 281.0 (233.0–328.0) vs. Pi*Z/Pi*ZZ: 251.0 (202.5–312.0), *p* = 0.016, Supplementary Table S2). Importantly, the prevalence of ACLD, dACLD and CSPH was significantly higher in patients with the PNPLA3 G-allele (ACLD: 30.0% vs. 19.1%, *p* < 0.001; dACLD: 7.7% vs. 4.3%, *p* = 0.012 b; CSPH: 18.0% vs. 11.4%, *p* = 0.001), and there was a numeric trend towards more frequent HCC (2.1% vs. 0.8%, *p* = 0.063, Supplementary Table S3).

### 3.4. Association with Liver Disease

Logistic and linear regression analyses were used to assess the impact of the individual risk allele on disease severity while adjusting for age, BMI and alcohol consumption (Table 2). First, regression analyses were performed for each SNP individually, showing that the PNPLA3 G-allele remained independently associated with a higher LSM (adjusted B: 2.707 (1.435–3.979), *p* < 0.001) and higher odds of ACLD (adjusted odds ratio (aOR): 1.971 (1.448–2.681), *p* < 0.001) and CSPH (aOR: 1.685 (1.180–2.406), *p* = 0.004), independent of age, BMI and current/previous alcohol consumption. Second, while the SERPINA1 Z-allele was neither associated with LSM (adjusted B: 2.581 (−0.244–5.406), *p* = 0.073) nor with the presence of ACLD (aOR: 1.748 (0.925–3.307), *p* = 0.086), it was independently associated with higher odds of CSPH (aOR: 2.122 (1.067–4.218), *p* = 0.032). However, there were numeric trends towards a higher LSM and higher odds of ACLD. After additionally adjusting for both genotypes, the PNPLA3 G-allele was still independently associated with a higher LSM (adjusted B: 2.715 (1.444–3.986), *p* < 0.001) and higher odds of ACLD (aOR: 1.989 (1.461–2.709), *p* < 0.001) and CSPH (aOR: 1.707 (1.194–2.441), *p* = 0.002), while the SERPINA1 Z-allele was still not associated with LSM (adjusted B: 2.624 (−0.182–5.430), *p* = 0.067) or higher odds of ACLD (aOR: 1.832 (0.963–3.483), *p* = 0.065), but with higher odds of CSPH (aOR: 2.196 (1.103–4.371), *p* = 0.025). Finally, the presence of any risk allele was associated with ACLD (aOR: 1.955 (1.427–2.678), *p* < 0.001) and CSPH (aOR: 1.675 (1.161–2.415), *p* = 0.006), while the presence of both risk alleles further increased the likelihood of ACLD (aOR: 3.892 (1.561–9.706), *p* = 0.004) and CSPH (aOR: 4.282 (1.667–10.996), *p* = 0.003).

In addition, the PNPLA3 G-allele in the subgroup of patients with CSPH was significantly associated with a higher LSM (*p* = 0.017; Supplementary Table S4). Sensitivity analyses in the subgroup of patients with NAFLD and ALD revealed similar results (Supplementary Tables S5 and S6). In addition, using alcohol as a continuous variable and then also correcting for type 2 diabetes mellitus did not change the associations (Supplementary Tables S7 and S8).

## 4. Discussion

In this study, we investigated the impact of genotyping for *PNPLA3* and *SERPINA1* in clinical routine, and analyzed these two SNPs in a cohort of consecutively referred subjects with symptomatic and/or suspected FLD at the time of their first contact with a hepatologist at a tertiary center. Our data confirm the central role of *PNPLA3* across the whole spectrum ofhile *SERPINA1* risk alleles may be particularly relevant in the development of advanced fibrotic stages or for the decompensation of liver disease [21].

In recent studies, *PNPLA3* has been identified as the central risk allele influencing liver disease severity in NAFLD [22]. *PNPLA3* has not only been associated with the development of liver cirrhosis and HCC [4,23,24], but has also shown a stronger association when compared with other risk alleles (e.g., *SERPINA1*, *TM6SF2*, *MBOAT7* or *GCKR*) [9,25,26,27]. The recent findings by Strnad et al. [13] showed that the *SERPINA1 Z*-allele was associated with disease severity and an increased risk of developing cirrhosis in patients with NAFLD and ALD, which was particularly significant as the association was independent of *PNPLA3*, *TM6SF2* and *MBOAT7*, and was numerically stronger than that of other risk variants for the development of NAFLD/ALD cirrhosis.

As both of these variants seem to influence the progression of liver disease through different pathways, the question arose whether the strong association of *SERPINA1* could be validated in an independent cohort. In our study, in line with the studies by Strnad et al. [13] and Mandorfer et al. [28], *SERPINA1* was associated with CSPH, but not with ACLD or LSM as a linear variable. Several factors may explain these observations: *SERPINA1* variants are significantly less common in the general population, which might diminish their influence on disease severity at a population level, despite its strong biological effect. Hence, our data that CSPH accumulates in patients with the *SERPINA1 Z*-allele confirm that this risk variant indeed has an influence on the progression of fibrosis at advanced stages of liver disease. This seems to be further pronounced if both risk variants (*SERPINA1* and *PNPLA3*) are present. Additionally, our findings suggest that the interpretation of genetic data depends on the composition of the patient cohorts from which these data are derived. While previous studies represent selected cohorts of patients undergoing liver biopsy to confirm suspicion of potential advanced liver disease, we present an unselected cohort of all consecutive patients with FLD being referred to a tertiary center for the first time, thus exhibiting less advanced CLD. Of note, it is well-known that differences in the frequencies of PNPLA3 variants translate into differences in the prevalence of FLD, which is particularly evident in groups with a Hispanic origin, who have a high carrier rate of the variant allele and thus a high prevalence of FLD, while the opposite is true for subjects from an African background [29]. Our findings are from a cohort of patients almost exclusively with a Caucasian background, and suggest that this variant is linked to liver disease prevalence at a population level.

Our clinical findings are also well in alignment with the proposed biological effects of the genetic variants. PNPLA3 variant predominantly increases the triglyceride content, and its effect increases with the presence of metabolic comorbidities or additional causes of liver disease [30]. Over time, this likely increases lipotoxic and oxidative stress, resulting in higher rates of advanced liver disease, including HCC, in carriers [24]. On the other hand, the SERPINA1 Z-allele exerts its adverse biological effects by augmenting proteotoxicity in the endoplasmic reticulum, which may be particularly relevant in fibrogenic pathways as liver disease progresses to more advanced stages, while it is compensated for at earlier stages [31,32].

This study has several limitations. To begin with, it is a cross-sectional retrospective study showing only a single time point in the natural course of liver disease, and hence, longitudinal data on liver-related morbidity and mortality are needed to support our findings. However, as genetic markers remain unchanged after randomization at inception, the observed association reflects the impact of the variant over the entire observed lifetime. Unfortunately, lifestyle factors such as diet and physical exercise were not evaluated, and might have influenced the conclusions of this study. Interestingly, Hamesch et al. [33] and Schneider et al. [34] showed that CAP values were higher in patients with homozygous *SERPINA1 Z/Z* variants while there was a numeric trend towards higher LSM values and higher odds of ACLD in carriers of the *SERPINA1* Z-allele in our cohort. However, our results might have benefited from a larger sample size, as the effect of even a biologically strong variant may be minor at a population level because of a lower prevalence, and hence, *SERPINA1* might be underpowered in our study cohort. In addition, our findings require validation in similar cohorts where genotyping is broadly used at the first visit.

Our findings raise important questions regarding the use of these genetic variants in a clinical setting as these variants are associated not only with disease severity in a cross-sectional manner, but also with the natural course of liver disease, including increased risk of ACLD, risk of decompensation and CSPH, higher rate of HCC and worse survival prospects [5,12,13,21,28]. Thus, improved risk stratification and individualised follow-up for the early identification of patients specifically at risk of developing significant liver disease may be cost-effective strategies. Although this cannot be quantified at this point in time, our findings support the inclusion of genetic variants into routine patient management and prospective evaluation because of its cost-effectiveness.

## 5. Conclusions

In conclusion, we provide data on the usefulness of genotyping for important risk alleles in a cohort of CLD in clinical routine patients with a Caucasian background. Specifically, we confirm the strong association between *PNPLA3* and liver disease severity. Although less evident at a population-based level, *SERPINA1* provides additional information on the risk of disease progression, particularly at advanced stages. Our findings suggest that genotyping for both variants is reasonable in the routine comprehensive assessment of patients with CLD.

## Figures and Tables

**Table 1:** Clinical and laboratory characterizations of the overall cohort (*n* = 1257).

Characteristics		Overall *n* = 1257 (100%)		
Clinical Data		Laboratory Measurements		
Age, years	52.7 ± 15.2	Bilirubin, mg/dL	0.6 (0.4–0.9)	
Male sex	759 (60.4%)	Albumin, g/dL	45.1 ± 4	
BMI, kg/m^2^	27.2 ± 5	INR	1.2 (1.2–1.2)	
LSM, kPa	5.9 (4.5–9.2)	Creatinine, mg/dL	0.9 (0.8–1)	
LSM ≥ 10 kPa	286 (22.8%)	Na, mmol/L	139.7 ± 2.5	
LSM ≥ 15 kPa	176 (14%)	Platelets, ×10^9^/L	237 (195–283)	
LSM ≥ 20 kPa	144 (11.5%)	Bilirubin, mg/dL	0.6 (0.4–0.9)	
CAP, dB	277 ± 68	AST (U/L)	33 (26–47)	
ACLD	309 (24.6%)	ALT(U/L)	40 (27–64)	
CSPH	185 (14.7%)	GGT (U/L)	70 (32–157)	
dACLD	76 (6%)	White-cell count, ×10^9^/L	6.3 (5.3–7.7)	
HCC	18 (1.4%)	CRP, mg/dL	0.2 (0.1–0.5)	
Child–Pugh Score		5 ± 1		
MELD Score		9.5 ± 2.3		
Fib-4 Score		1.14 (0.76–1.80)		
*Etiology of liver disease*				
NAFLD		1048 (83.4%)		
ALD		209 (16.6%)		
*Genotypes*		*χ* ^2^	*p*	*q*
PNPLA3 C/C	623 (49.6%)	7.1308	0.6925	0.3075
PNPLA3 G/C	495 (39.4%)			
PNPLA3 G/G	139 (11.1%)			
SERPINA1 M/M	1147 (91.2%)	0.0074	0.9730	0.0270
SERPINA1 M/S	43 (3.4%)			
SERPINA1 M/Z	66 (5.3%)			
SERPINA1 Z/Z	1 (0.1%)			

ACLD—advanced chronic liver disease;dACLD—decompensated ACLD; ALD—alcoholic liver disease; ALT—alanine transferase; AST—aspartate aminotransferase; BMI—body mass index; CAP—controlled attenuation parameter; CRP—C-reactive protein; CSPH—clinically significant portal hypertension; HCC—hepatocellular carcinoma; GGT—gamma-glutamyl transferase; LSM—liver stiffness measurement; NAFLD—non-alcoholic fatty liver disease.

**Table 2:** Multivariable linear and logistic regression analyses investigating the association of individual risk alleles with the liver stiffness measurement (LSM), the presence of advanced chronic liver disease (ACLD) and clinically significant portal hypertension (CSPH): (**A**) for the *PNPLA3 G*-allele and its covariables, (**B**) for the *SERPINA1 Z*-allele and it covariables and (**C**) in a combined multivariable regression model for both the *PNPLA3 G*-allele and *SERPINA1 Z*-allele. (**D**) Multivariable logistic regression model comparing patients with no risk allele (reference) to those with one or two risk alleles. Covariables were: age (per year), BMI (per kg/m^2^) and active or past alcohol abuse (≥2 drinks per day for women, and ≥3 drinks per day for men).

**A**	**LSM, kPa**	**ACLD**	**CSPH**
Age, year	0.114 (0.072–0.157), *p* < 0.001	1.055 (1.042–1.068), *p* < 0.001	1.042 (1.028–1.056), *p* < 0.001
BMI, kg/m^2^	0.327 (0.199–0.454), *p* < 0.001	1.124 (1.089–1.160), *p* < 0.001	1.053 (1.017–1.091), *p* = 0.004
Alcohol abuse	13.224 (11.580–14.867), *p* < 0.001	7.718 (5.457–10.915), *p* < 0.001	7.280 (5.082–10.428), *p* < 0.001
*PNPLA3 G*-allele	2.707 (1.435–3.979), *p* < 0.001	1.971 (1.448–2.681), *p* < 0.001	1.685 (1.180–2.406), *p* = 0.004
**B**	**LSM, kPa**	**ACLD**	**CSPH**
Age, year	0.111 (0.068–0.154), *p* < 0.001	1.053 (1.041–1.065), *p* < 0.001	1.041 (1.027–1.055), *p* < 0.001
BMI, kg/m^2^	0.348 (0.220–0.476), *p* < 0.001	1.127 (1.092–1.163), *p* < 0.001	1.056 (1.020–1.094), *p* = 0.002
Alcohol abuse	13.462 (11.809–15.116), *p* < 0.001	7.851 (5.563–11.081), *p* < 0.001	7.598 (5.297–10.900), *p* < 0.001
*SERPINA1 Z*-allele	2.581 (−0.244–5.406), *p* = 0.073	1.748 (0.925–3.307), *p* = 0.086	2.122 (1.067–4.218), *p* = 0.032
**C**	**LSM, kPa**	**ACLD**	**CSPH**
Age, year	0.113 (0.070–0.156), *p* < 0.001	1.055 (1.042–1.067), *p* < 0.001	1.042 (1.028–1.056), *p* < 0.001
BMI, kg/m^2^	0.332 (0.205–0.459), *p* < 0.001	1.125 (1.090–1.161), *p* < 0.001	1.054 (1.018–1.092), *p* = 0.003
Alcohol abuse	13.302 (11.658–14.947), *p* < 0.001	7.896 (5.573–11.188), *p* < 0.001	7.553 (5.252–10.862), *p* < 0.001
*PNPLA3 G*-allele	2.715 (1.444–3.986), *p* < 0.001	1.989 (1.461–2.709), *p* < 0.001	1.707 (1.194–2.441), *p* = 0.003
*SERPINA1 Z*-allele	2.624 (−0.182–5.430), *p* = 0.067	1.832 (0.963–3.483), *p* = 0.065	2.196 (1.103–4.371), *p* = 0.025
**D**	**ACLD**		**CSPH**
Age, year	1.054 (1.042–1.067), *p* < 0.001		1.042 (1.028–1.056), *p* < 0.001
BMI, kg/m^2^	1.125 (1.091–1.161), *p* < 0.001		1.054 (1.018–1.092), *p* = 0.003
Alcohol abuse	7.918 (5.589–11.216), *p* < 0.001		7.559 (5.258–10.866), *p* < 0.001
No risk allele	Reference		Reference
One risk allele	1.955 (1.427–2.678), *p* < 0.001		1.675 (1.161–2.415), *p* = 0.006
Two risk alleles	3.892 (1.561–9.706), *p* = 0.004		4.282 (1.667–10.996), *p* = 0.003

ACLD—advanced chronic liver disease; BMI—body mass index; CSPH—clinically significant portal hypertension; LSM—liver stiffness measurement.

## Data Availability

The data are available from the authors at request.

## Supplementary Materials

The following are available online at https://www.mdpi.com/2075-4426/11/3/165/s1, Supplementary Table S1: Patient and disease characteristics compared among carriers and non-carriers of the PNPLA3 G-allele and SERPINA1 Z-allele. Supplementary Table S2. Comparison of liver stiffness measurement (LSM), controlled attenuation parameter (CAP), Child-Pugh-score (CPS), MELD score and Fib-4 score among patients with and without the respective risk alleles. Supplementary Table S3. Comparison of prevalence of hepatocellular carcinoma HCC), advanced chronic liver disease (ACLD), decompensated ACLD (dACLD), and clinically significant portal hypertension (CSPH) in patients with and without the respective risk allele. Supplementary Table S4. Comparison of liver stiffness measurement (LSM), controlled attenuation parameter (CAP), Child-Pugh-score (CPS), MELD score and Fib-4 score among patients with and without the respective risk alleles when only considering patients with ACLD or CSPH. Supplementary Table S5. Linear and logistic regression analyses investigating the association of individual risk alleles with liver stiffness measurement (LSM), presence of advanced chronic liver disease (ACLD) and clinically significant portal hypertension (CSPH) in patients with non-alcoholic fatty liver disease (*n* = 1048) only: (A) for PNPLA3 and covariables, (B) for SERPINA1 and covariables and (C) in a combined regression model for both PNPLA3 and SERPINA1. (D) Logistic regression model comparing patients with no risk allele (reference) to those with any and two risk alleles. Covariates were: Age (per year) and BMI (per kg/m^2^). Supplementary Table S6. Linear and logistic regression analyses investigating the association of individual risk alleles with liver stiffness measurement (LSM), presence of advanced chronic liver disease (ACLD) and clinically significant portal hypertension (CSPH) in patients with alcoholic liver disease (*n* = 209) only: (A) for PNPLA3 and covariables, (B) for SERPINA1 and covariables and (C) in a combined regression model for both PNPLA3 and SERPINA1. (D) Logistic regression model comparing patients with no risk allele (reference) to those with any and two risk alleles. Covariates were: Age (per year), BMI (per kg/m^2^), active or past alcohol abuse (women ≥ 2 drinks per day, men ≥ 3 drinks per day). Supplementary Table S7. Multivariable linear and logistic regression analyses investigating the association of individual risk alleles with liver stiffness measurement (LSM), presence of advanced chronic liver disease (ACLD) and clinically significant portal hypertension (CSPH): (A) for PNPLA3 and covariables, (B) for SERPINA1 and covariables and (C) in a combined regression model for both PNPLA3 and SERPINA1. (D) Logistic regression model comparing patients with no risk allele (reference) to those with any and two risk alleles. Covariables were: Age (per year), BMI (per kg/m^2^), alcohol consumption (per drink per day). Supplementary Table S8. Multivariable linear and logistic regression analyses investigating the association of individual risk alleles with liver stiffness measurement (LSM), presence of advanced chronic liver disease (ACLD) and clinically significant portal hypertension (CSPH): (A) for PNPLA3 and covariables, (B) for SERPINA1 and covariables and (C) in a combined multivariable regression model for both PNPLA3 and SERPINA1. (D) Multivariable logistic regression model comparing patients with no risk allele (reference) to those with any and two risk alleles. Covariables were: Age (per year), BMI (per kg/m^2^), active or past alcohol abuse (women ≥ 2 drinks per day, men ≥ 3 drinks per day), type 2 diabetes mellitus.

## References

[1] Sookoian S., Pirola C.J. (2011). Meta-analysis of the influence of I148M variant of patatin-like phospholipase domain containing 3 gene (PNPLA3) on the susceptibility and histological severity of nonalcoholic fatty liver disease. Hepatology.

[2] Romeo S., Kozlitina J., Xing C., Pertsemlidis A., Cox D., Pennacchio L.A., Boerwinkle E., Cohen J.C., Hobbs H.H. (2008). Genetic variation in PNPLA3 confers susceptibility to nonalcoholic fatty liver disease. Nat. Genet..

[3] Buch S., Stickel F., Trépo E., Way M., Herrmann A., Nischalke H.D., Brosch M., Rosendahl J., Berg T., Ridinger M. (2015). A genome-wide association study confirms PNPLA3 and identifies TM6SF2 and MBOAT7 as risk loci for alcohol-related cirrhosis. Nat. Genet..

[4] Salameh H., Raff E., Erwin A., Seth D., Nischalke H.D., Falleti E., Burza M.A., Leathert J., Romeo S., Molinaro A. (2015). PNPLA3 Gene Polymorphism Is Associated With Predisposition to and Severity of Alcoholic Liver Disease. Am. J. Gastroenterol..

[5] Stickel F., Buch S., Lau K., Meyer zu Schwabedissen H., Berg T., Ridinger M., Rietschel M., Schafmayer C., Braun F., Hinrichsen H. (2011). Genetic variation in the PNPLA3 gene is associated with alcoholic liver injury in caucasians. Hepatology.

[6] Trépo E., Gustot T., Degré D., Lemmers A., Verset L., Demetter P., Ouziel R., Quertinmont E., Vercruysse V., Amininejad L. (2011). Common polymorphism in the PNPLA3/adiponutrin gene confers higher risk of cirrhosis and liver damage in alcoholic liver disease. J. Hepatol..

[7] Atkinson S.R., Way M.J., McQuillin A., Morgan M.Y., Thursz M.R. (2017). Homozygosity for rs738409:G in PNPLA3 is associated with increased mortality following an episode of severe alcoholic hepatitis. J. Hepatol..

[8] Stättermayer A.F., Scherzer T., Beinhardt S., Rutter K., Hofer H., Ferenci P. (2014). Review article: Genetic factors that modify the outcome of viral hepatitis. Aliment Pharm..

[9] Dongiovanni P., Romeo S., Valenti L. (2015). Genetic Factors in the Pathogenesis of Nonalcoholic Fatty Liver and Steatohepatitis. Biomed. Res. Int..

[10] Unalp-Arida A., Ruhl C.E. (2020). Patatin-Like Phospholipase Domain-Containing Protein 3 I148M and Liver Fat and Fibrosis Scores Predict Liver Disease Mortality in the U.S. Population. Hepatology.

[11] Fischer H.-P., Ortiz-Pallardó M.E., Ko Y., Esch C., Zhou H. (2000). Chronic liver disease in heterozygous α1-antitrypsin deficiency PiZ. J. Hepatol..

[12] Luukkonen P.K., Salomaa V., Åberg F. (2020). The Pi*MZ Allele in Alpha-1 Antitrypsin Increases Liver-Related Outcomes in a Population-Based Study. Gastroenterology.

[13] Strnad P., Buch S., Hamesch K., Fischer J., Rosendahl J., Schmelz R., Brueckner S., Brosch M., Heimes C.V., Woditsch V. (2019). Heterozygous carriage of the alpha1-antitrypsin Pi*Z variant increases the risk to develop liver cirrhosis. Gut.

[14] Abul-Husn N.S., Cheng X., Li A.H., Xin Y., Schurmann C., Stevis P., Liu Y., Kozlitina J., Stender S., Wood G.C. (2018). A Protein-Truncating HSD17B13 Variant and Protection from Chronic Liver Disease. N. Engl. J. Med..

[15] (2016). EASL-EASD-EASO Clinical Practice Guidelines for the management of non-alcoholic fatty liver disease. J. Hepatol..

[16] Pons M., Rodríguez-Tajes S., Esteban J.I., Mariño Z., Vargas V., Lens S., Buti M., Augustin S., Forns X., Mínguez B. (2020). Non-invasive prediction of liver-related events in patients with HCV-associated compensated advanced chronic liver disease after oral antivirals. J. Hepatol..

[17] Semmler G., Wöran K., Scheiner B., Unger L.W., Paternostro R., Stift J., Schwabl P., Bucsics T., Bauer D., Simbrunner B. (2020). Novel reliability criteria for controlled attenuation parameter assessments for non-invasive evaluation of hepatic steatosis. United Eur. Gastroenterol. J..

[18] Boursier J., Zarski J.P., de Ledinghen V., Rousselet M.C., Sturm N., Lebail B., Fouchard-Hubert I., Gallois Y., Oberti F., Bertrais S. (2013). Determination of reliability criteria for liver stiffness evaluation by transient elastography. Hepatology.

[19] Kedenko L., Lamina C., Kedenko I., Kollerits B., Kiesslich T., Iglseder B., Kronenberg F., Paulweber B. (2014). Genetic polymorphisms at SIRT1 and FOXO1 are associated with carotid atherosclerosis in the SAPHIR cohort. BMC Med. Genet..

[20] Rodriguez S., Gaunt T.R., Day I.N. (2009). Hardy-Weinberg equilibrium testing of biological ascertainment for Mendelian randomization studies. Am. J. Epidemiol..

[21] Schaefer B., Mandorfer M., Viveiros A., Finkenstedt A., Ferenci P., Schneeberger S., Tilg H., Zoller H. (2018). Heterozygosity for the alpha-1-antitrypsin Z allele in cirrhosis is associated with more advanced disease. Liver Transpl..

[22] Krawczyk M., Liebe R., Lammert F. (2020). Toward Genetic Prediction of Nonalcoholic Fatty Liver Disease Trajectories: PNPLA3 and Beyond. Gastroenterology.

[23] Hassan M.M., Kaseb A., Etzel C.J., El-Serag H., Spitz M.R., Chang P., Hale K.S., Liu M., Rashid A., Shama M. (2013). Genetic variation in the PNPLA3 gene and hepatocellular carcinoma in USA: Risk and prognosis prediction. Mol. Carcinog..

[24] Trépo E., Romeo S., Zucman-Rossi J., Nahon P. (2016). PNPLA3 gene in liver diseases. J. Hepatol..

[25] Bianco C., Jamialahmadi O., Pelusi S., Baselli G., Dongiovanni P., Zanoni I., Santoro L., Maier S., Liguori A., Meroni M. (2020). Non-invasive stratification of hepatocellular carcinoma risk in non-alcoholic fatty liver using polygenic risk scores. J. Hepatol..

[26] Kawaguchi T., Shima T., Mizuno M., Mitsumoto Y., Umemura A., Kanbara Y., Tanaka S., Sumida Y., Yasui K., Takahashi M. (2018). Risk estimation model for nonalcoholic fatty liver disease in the Japanese using multiple genetic markers. PLoS ONE.

[27] León-Mimila P., Vega-Badillo J., Gutiérrez-Vidal R., Villamil-Ramírez H., Villareal-Molina T., Larrieta-Carrasco E., López-Contreras B.E., Kauffer L.R., Maldonado-Pintado D.G., Méndez-Sánchez N. (2015). A genetic risk score is associated with hepatic triglyceride content and non-alcoholic steatohepatitis in Mexicans with morbid obesity. Exp. Mol. Pathol..

[28] Mandorfer M., Bucsics T., Hutya V., Schmid-Scherzer K., Schaefer B., Zoller H., Ferlitsch A., Peck-Radosavljevic M., Trauner M., Ferenci P. (2018). Liver disease in adults with α1-antitrypsin deficiency. United Eur. Gastroenterol. J..

[29] Wagenknecht L.E., Palmer N.D., Bowden D.W., Rotter J.I., Norris J.M., Ziegler J., Chen Y.D., Haffner S., Scherzinger A., Langefeld C.D. (2011). Association of PNPLA3 with non-alcoholic fatty liver disease in a minority cohort: The Insulin Resistance Atherosclerosis Family Study. Liver Int. Off. J. Int. Assoc. Study Liver.

[30] Pingitore P., Romeo S. (2019). The role of PNPLA3 in health and disease. Biochim. Et Biophys. Acta. Mol. Cell Biol. Lipids.

[31] Teckman J.H., An J.K., Blomenkamp K., Schmidt B., Perlmutter D. (2004). Mitochondrial autophagy and injury in the liver in alpha 1-antitrypsin deficiency. Am. J. Physiol. Gastrointest. Liver Physiol..

[32] Strnad P., McElvaney N.G., Lomas D.A. (2020). Alpha(1)-Antitrypsin Deficiency. N. Engl. J. Med..

[33] Hamesch K., Mandorfer M., Pereira V.M., Moeller L.S., Pons M., Dolman G.E., Reichert M.C., Schneider C.V., Woditsch V., Voss J. (2019). Liver Fibrosis and Metabolic Alterations in Adults With alpha-1-antitrypsin Deficiency Caused by the Pi*ZZ Mutation. Gastroenterology.

[34] Schneider C.V., Hamesch K., Gross A., Mandorfer M., Moeller L.S., Pereira V., Pons M., Kuca P., Reichert M.C., Benini F. (2020). Liver Phenotypes of European Adults Heterozygous or Homozygous for Pi∗Z Variant of AAT (Pi∗MZ vs. Pi∗ZZ genotype) and Noncarriers. Gastroenterology.

