# Supplementary material for: PNPLA3 and SERPINA1 Variants Are Associated with Severity of Fatty Liver Disease at First Referral to a Tertiary Center
Source: J Pers Med. 2021 Mar 1;11(3):165. doi: 10.3390/jpm11030165 (PMC7999282; doi:10.3390/jpm11030165)
Supplement: Supplementary file 1 [file jpm-11-00165-s001.pdf]

SUPPLEMENT

Supplementary Table-1

	PNPLA3 C/C n=623	PNPLA3 C/G or G/G n=634	*P*	SERPINA1 M/M or M/S n=1190	SERPINA1 M/Z or Z/Z n=67	*P*
Age, years	52.8±15.4	52.6±15.0	0.877	52.6±15.1	54.1±15.8	0.441
Male sex,	377 (60.5%)	382 (60.3%)	0.925	730 (61.3%)	29 (43.3%)	**0.003**
BMI, kg/m^2	26.2±4.8	27.5±5.2	0.053	27.2±5.0	26.3±5.4	0.134
Bilirubin, mg/dL	0.6 (0.4-0.9)	0.6 (0.4-0.9)	0.164	0.6 (0.4-0.9)	0.7 (0.4-1.0)	0.307
Albumin, g/dL	45.0±4.1	45.2±4.0	0.491	45.1±4.0	45.5±4.6	0.385
INR	1.2 (1.2-1.2)	1.2 (1.2-1.2)	0.120	1.2 (1.2-1.2)	1.2 (1.2-1.2)	0.146
Creatinine, mg/dL	0.9 (0.8-1.0)	0.9 (0.8-1.0)	0.916	0.9 (0.8-1.0)	0.9 (0.8-1.0)	0.252
Na, mmol/L	139.7±2.4	139.6±2.7	0.537	139.7±2.5	139.1±2.7	0.077
Platelets, x10^9/L	239.5 (203.0-282.0)	235.0 (188.0-285.0)	0.176	238.0 (195.0-283.0)	220.0 (188.0-286.0)	0.441
White-cell count, x10^9/L	6.3 (5.3-7.6)	6.4 (5.3-7.8)	0.792	6.4 (5.3-7.7)	6.0 (5.1-7.8)	0.246
CRP, mg/dL	0.2 (0.1-0.4)	0.2 (0.1-0.5)	0.062	0.2 (0.1-0.5)	0.2 (0.1-0.6)	0.635
AP, U/L	76.0 (61.0-95.5)	75.0 (60.0-97.0)	0.980	75.5 (61.0-96.0)	75.0 (53.0-101.0)	0.249
AP/ULN	0.73 (0.59-0.92)	0.72 (0.58-0.93)	0.980	0.73 (0.59-0.92)	0.72 (0.51-0.97)	0.249
AST, U/L	31.0 (24.0-43.0)	35.0 (26.0-52.8)	**<0.001**	34.0 (26.0-48.0)	33.0 (26.0-44.3)	0.614
AST/ULN	0.83 (0.65-1.10)	0.93 (0.71-1.37)	**<0.001**	0.88 (0.68-1.23)	0.85 (0.70-1.23)	0.895
ALT, U/L	38.0 (25.0-59.0)	44.0 (28.0-72.0)	**0.001**	41.0 (27.0-65.0)	36.0 (24.0-55.0)	0.096
ALT/ULN	0.86 (0.60-1.31)	1.00 (0.66-1.61)	**0.001**	0.94 (0.63-1.46)	0.82 (0.64-1.22)	0.351
GGT, U/L	70.0 (31.5-147.5)	71.5 (34.0-169.3)	0.626	72.0 (33.0-159.8)	62.0 (30.0-134.0)	0.218
GGT/ULN	0.50 (0.01-1.93)	0.60 (0.01-1.85)	0.388	0.58 (0.01-1.95)	0.01 (0.01-1.10)	**0.009**
Cholinesterase, U/L	8.3 (7.0-9.5)	8.2 (6.7-9.4)	0.241	8.3 (6.8-9.5)	7.5 (6.2-8.5)	**0.003**

Supplementary Table-1. Patient and disease characteristics compared among carriers and non-carriers of the *PNPLA3 G-allele* and *SERPINA1 Z-allele.* The ULN was defined as follows: AP ≥ 104 U/L; AST ≥ 40 U/L for men/≥ 35 U/L for women; ALT ≥ 50 U/L for men/≥ 35 for women, GGT ≥ 60 for men/≥ 40 for women.

Abbreviations:

ALT alanine transferase
AST aspartate aminotransferase
CRP C-reactive protein
GGT gamma-glutamyl transferase
ULN upper limit of normal

Supplementary Table-2

Genotypes	LSM, kPa	CAP, dB/m	CPS	MELD	Fib-4
**PNPLA3 C/C n=623**	5.8 (4.5-8.0)	275.5 (224.0-324.3)	**5.2±0.6**	9.4±.21	1.1 (0.7-1.7)
**PNPLA3 G/C or G/G n=634**	6.1 (4.6-11.2)	285.0 (236.0-330.3)	5.2±0.7	9.6±2.4	1.2 (0.8-2.0)
P	**0.002**	0.083	0.131	0.331	**0.036**
**SERPINA1 M/M or M/S n=1190**	5.9 (4.5-9.1)	281.0 (233.0-328.0)	5.2±0.6	9.5±2.3	1.1 (0.8-1.8)
**SERPINA1 M/Z or Z/Z n=67**	6.0 (4.4-11.0)	251.0 (202.5-312.0)	5.3±0.9	9.5±2.2	1.2 (0.7-1.8)
P	0.979	**0.016**	0.235	0.975	0.773
**No risk allele n=588**	5.8 (4.4-8.0)	279.0 (226.0-326.0)	5.2±0.6	9.4±2.1	1.1 (0.7-1.7)
**One risk allele n=637**	6.1 (4.6-11.1)	283.0 (235.0-329.0)	5.2±0.7	9.6±2.4	1.2 (0.8-2.0)
**Two risk alleles n=32**	6.4 (4.2-13.1)	279.0 (217.5-333.3)	5.4±1.1	9.3±2.0	1.1 (0.7-1.7)
P	**0.008**	0.516	0.100	0.397	0.062

Supplementary Table-2. Comparison of liver stiffness measurement (LSM), controlled attenuation parameter (CAP), Child-Pugh-score (CPS), MELD score and Fib-4 score among patients with and without the respective risk alleles.

Abbreviations:

CAP controlled attenuation parameter
CPS Child-Pugh score
IQR interquartile range
LSM liver stiffness measurement

Supplementary Table-3

Genotypes	**HCC**	**ACLD**	**dACLD**	**CSPH**	***P***			
**PNPLA3 C/C n=623**	n=5 (0.8%)	n=119 (19.1%)	n=27 (4.3%)	n=71 (11.4%)	0.063	**<0.001**	**0.012**	**0.001**
**PNPLA3 G/C or G/G n=634**	n=13 (2.1%)	n=190 (30.0%)	n=49 (7.7%)	n=114 (18.0%)				
**SERPINA1 M/M or M/S n=1190**	n=16 (1.3%)	n=289 (24.3%)	n=70 (5.9%)	n=171 (14.4%)	0.249	0.303	0.289	0.142
**SERPINA1 M/Z or Z/Z n=67**	n=2 (3.0%)	n=20 (29.9%)	n=6 (9.0%)	n=14 (20.9%)				

Supplementary Table-3. Comparison of prevalence of hepatocellular carcinoma HCC), advanced chronic liver disease (ACLD), decompensated ACLD (dACLD), and clinically significant portal hypertension (CSPH) in patients with and without the respective risk allele.

Abbreviations:

ACLD advanced chronic liver disease
dACLD decompensated ACLD
CSPH clinically significant portal hypertension
HCC hepatocellular carcinoma

Supplementary Table-4

	LSM, kPa	CAP, dB/m	CPS	MELD	Fib-4
ACLD					
**PNPLA3 C/C n=119**	16.3 (11.6-29.7)	305.0 (262.0-342.5)	5.6±1.0	10.4±3.1	2.3 (1.3-4.1)
**PNPLA3 G/C or G/G n=190**	18.5 (12.0-35.3)	306.0 (261.3-350.8)	5.6±1.1	10.3±3.0	2.6 (1.5-3.9)
P	0.060	0.784	0.944	0.922	0.582
CSPH					
**PNPLA3 C/C n=71**	25.7 (16.3-39.6)	293,0 (243.5-329.0)	5.9±1.2	11.2±3.6	2.8 (1.4-5.1)
**PNPLA3 G/C or G/G n=114**	32.5 (21.7-53.9)	295.0 (249.3-342.0)	5.8±1.3	10.9±3.4	3.0 (1.8-4.7)
P	**0.017**	0.573	0.814	0.530	0.628
ACLD					
**SERPINA1 M/M or M/S n=289**	17.0 (11.8-34.3)	306.0 (264.0-351.0)	5.6±1.1	10.4±3.1	2.5 (1.5-4.0)
**SERPINA1 M/Z or Z/Z n=20**	22.7 (11.2-36.5)	274.0 (229.0-333.0)	5.7±1.4	9.8±2.4	3.3 (1.6-5.1)
P	0.736	0.063	0.743	0.401	0.354
CSPH					
**SERPINA1 M/M or M/S n=171**	29.6 (20.9-48.0)	295.5 (250.0-333.3)	5.9±1.2	11.1±3.5	2.9 (1.6-4.8)
**SERPINA1 M/Z or Z/Z n=14**	32.0 (19.0-43.3)	260.0 (229.0-341.0)	5.9±1.6	10.3±2.7	3.4 (1.7-5.2)
P	0.985	0.460	0.994	0.454	0.681

Supplementary Table-4. Comparison of liver stiffness measurement (LSM), controlled attenuation parameter (CAP), Child-Pugh-score (CPS), MELD score and Fib-4 score among patients with and without the respective risk alleles when only considering patients with ACLD or CSPH.

Abbreviations:
ACLD advanced chronic liver disease
CAP controlled attenuation parameter
CPS Child-Pugh score
CSPH clinically significant portal hypertension
LSM liver stiffness measurement

Supplementary Table-5

A	LSM, kPa	ACLD	CSPH
**Age, year**	0.112 (0.080-0.143)<br>*P*<**0.001**	1.061 (1.046-1.076)<br>*P*<**0.001**	1.060 (1.041-1.078)<br>*P*<**0.001**
**BMI, kg/m²**	0.445 (0.347-0.542)<br>*P*<**0.001**	1.181 (1.138-1.226)<br>*P*<**0.001**	1.125 (1.077-1.175)<br>*P*<**0.001**
**PNPLA3 G-allele**	1.324 (0.353-2.294)<br>*P*=**0.008**	2.029 (1.405-2.929)<br>*P*<**0.001**	1.843 (1.162-2.926)<br>*P*=**0.009**

B	LSM, kPa	ACLD	CSPH
**Age, year**	0.109 (0.078-0.141)<br>*P*<**0.001**	1.059 (1.044-1.073)<br>*P*<**0.001**	1.058 (1.040-1.077)<br>*P*<**0.001**
**BMI, kg/m²**	0.459 (0.361-0.556)<br>*P*<**0.001**	1.184 (1.141-1.229)<br>*P*<**0.001**	1.129 (1.082-1.179)<br>*P*<**0.001**
**SERPINA1 Z-allele**	1.899 (-0.180-3.977)<br>*P*=0.073	1.478 (0.710-3.075)<br>*P*=0.296	1.924 (0.859-4.311)<br>*P*=0.112

C	LSM, kPa	ACLD	CSPH
**Age, year**	0.111 (0.079-0.142)<br>*P*<**0.001**	1.061 (1.046-1.076)<br>*P*<**0.001**	1.059 (1.040-1.078)<br>*P*<**0.001**
**BMI, kg/m²**	0.450 (0.352-0.547)<br>*P*<**0.001**	1.182 (1.138-1.227)<br>*P*<**0.001**	1.125 (1.078-1.175)<br>*P*<**0.001**
**PNPLA3 G-allele**	1.318 (0.349-2.287)<br>*P*=**0.008**	2.039 (1.411-2.946)<br>*P*<**0.001**	1.858 (1.169-2.953)<br>*P*=**0.009**
**SERPINA1 Z-allele**	1.880 (-0.192-3.952)<br>*P*=0.075	1.529 (0.730-3.201)<br>*P*=0.260	1.974 (0.875-4.452)<br>*P*=0.101

D	ACLD	CSPH
**Age, year**	1.060 (1.046-1.075)<br>*P*<**0.001**	1.059 (1.041-1.078)<br>*P*<**0.001**
**BMI, kg/m²**	1.183 (1.139-1.228)<br>*P*<**0.001**	1.126 (1.078-1.176)<br>*P*<**0.001**
**No risk allele**	Reference	Reference
**One risk allele**	1.871 (1.286-2.722)<br>*P*=**0.001**	1.769 (1.098-2.852)<br>*P*=**0.019**
**Two risk alleles**	4.212 (1.558-11.385)<br>*P*=**0.005**	4.278 (1.453-12.596)<br>*P*=**0.008**

Supplementary Table-5. Linear and logistic regression analyses investigating the association of individual risk alleles with liver stiffness measurement (LSM), presence of advanced chronic liver disease (ACLD) and clinically significant portal hypertension (CSPH) in patients with non-alcoholic fatty liver disease (n=1048) only: (**A**) for PNPLA3 and covariables, (**B**) for SERPINA1 and covariables and (**C**) in a combined regression model for both PNPLA3 and SERPINA1. (**D**) Logistic regression

model comparing patients with no risk allele (reference) to those with any and two risk alleles. Covariates were: Age (per year) and BMI (per kg/m^2).

Abbreviations:

ACLD advanced chronic liver disease
BMI body mass index
CSPH clinically significant portal hypertension
LSM liver stiffness measurement

Supplementary Table-6

A	LSM, kPa	ACLD	CSPH
**Age, year**	0.126 (-0.126-0.377)<br>*P*=0.326	1.051 (1.023-1.081)<br>*P*<**0.001**	1.015 (0.991-1.040)<br>*P*=0.215
**BMI, kg/m²**	-0.073 (-0.656-0.509)<br>*P*=0.804	0.993 (0.936-1.054)<br>*P*=0.826	0.966 (0.912-1.022)<br>*P*=0.223
**PNPLA3 G-allele**	9.912 (3.931-15.893)<br>*P*=**0.001**	1.854 (1.016-3.383)<br>*P*=**0.044**	1.468 (0.833-2.588)<br>*P*=0.184

B	LSM, kPa	ACLD	CSPH
**Age, year**	0.134 (-0.124-0.391)<br>*P*=0.308	1.050 (1.021-1.079)<br>*P*=**0.001**	1.015 (0.991-1.039)<br>*P*=0.234
**BMI, kg/m²**	-0.057 (-0.654-0.540)<br>*P*=0.851	0.995 (0.938-1.054)<br>*P*=0.858	0.966 (0.912-1.022)<br>*P*=0.231
**SERPINA1 Z-allele**	7.929 (-8.747-24.604)<br>*P*=0.350	NE	3.113 (0.585-16.560)<br>*P*=0.183

C	LSM, kPa	ACLD	CSPH
**Age, year**	0.113 (-0.138-0.365)<br>*P*=0.376	1.050 (1.021-1.079)<br>*P*=**0.001**	1.014 (0.990-1.039)<br>*P*=0.259
**BMI, kg/m²**	-0.075 (-0.656-0.507)<br>*P*=0.800	0.995 (0.937-1.056)<br>*P*=0.857	0.965 (0.912-1.021)<br>*P*=0.220
**PNPLA3 G-allele**	10.312 (4.311-16.314)<br>*P*=**0.001**	1.997 (1.086-3.673)<br>*P*=**0.026**	1.544 (0.870-2.742)<br>*P*=0.138
**SERPINA1 Z-allele**	10.768 (-5.565-27.101)<br>*P*=0.195	NE	3.546 (0.655-19.185)<br>*P*=0.142

D	ACLD	CSPH
**Age, yr**	1.050 (1.022-1.080)<br>*P*=**0.001**	1.014 (0.990-1.039)<br>*P*=0.251
**BMI, kg/m²**	0.993 (0.936-1.054)<br>*P*=0.829	0.965 (0.912-1.021)<br>*P*=0.218
**No risk allele**	Reference	Reference
**One risk allele**	2.125 (1.159-3.897)<br>*P*=**0.015**	1.515 (0.853-2.690)<br>*P*=0.157
**Two risk alleles**	NE	NE

Supplementary Table-6. Linear and logistic regression analyses investigating the association of individual risk alleles with liver stiffness measurement (LSM), presence of advanced chronic liver disease (ACLD) and clinically significant portal hypertension (CSPH) in patients with alcoholic liver disease (n=209) only: (**A**) for PNPLA3 and covariables, (**B**) for SERPINA1 and covariables and (**C**) in a combined regression model for both PNPLA3 and SERPINA1. (**D**) Logistic regression model comparing patients with no risk allele (reference) to those with any and two risk

alleles. Covariates were: Age (per year), BMI (per kg/m^2), active or past alcohol abuse (women ≥ 2 drinks per day, men ≥ 3 drinks per day).

Abbreviations:

ACLD advanced chronic liver disease
BMI body mass index
CSPH clinically significant portal hypertension
LSM liver stiffness measurement
NE not evaluable

Supplementary Table-7

A	LSM, kPa	ACLD	CSPH
**Age, year**	0.157 (0.112-0.202) *P*<**0.001**	1.057 (1.045-1.068) *P*<**0.001**	1.046 (1.033-1.060) *P*<**0.001**
**BMI, kg/m²**	0.294 (0.159-0.429) *P*<**0.001**	1.104 (1.072-1.137) *P*<**0.001**	1.041 (1.007-1.077) *P*=**0.017**
**Alcohol consumption, drinks/d**	1.336 (1.044-1.628) *P*<**0.001**	1.206 (1.139-1.276) *P*<**0.001**	1.199 (1.130-1.273) *P*<**0.001**
**PNPLA3 G-allele**	3.124 (1.774-4.474) *P*<**0.001**	1.977 (1.477-2.644) *P*<**0.001**	1.742 (1.241-2.444) *P*=**0.001**

B	LSM, kPa	ACLD	CSPH
**Age, year**	0.155 (0.110-0.200) *P*<**0.001**	1.055 (1.043-1.066) *P*<**0.001**	1.046 (1.033-1.059) *P*<**0.001**
**BMI, kg/m²**	0.316 (0.180-0.452) *P*<**0.001**	1.106 (1.074-1.139) *P*<**0.001**	1.045 (1.011-1.080) *P*=**0.009**
**Alcohol consumption, drinks/d**	1.351 (1.056-1.646) *P*<**0.001**	1.205 (1.139-1.274) *P*<**0.001**	1.202 (1.133-1.276) *P*<**0.001**
**SERPINA1 Z-allele**	2.038 (-0.968-5.044) *P*=0.184	1.515 (0.828-2.772) *P*=0.178	1.764 (0.924-3.370) *P*=0.085

C	LSM, kPa	ACLD	CSPH
**Age, year**	0.156 (0.111-0.201) *P*<**0.001**	1.056 (1.045-1.068) *P*<**0.001**	1.046 (1.033-1.059) *P*<**0.001**
**BMI, kg/m²**	0.298 (0.163-0.433) *P*<**0.001**	1.105 (1.073-1.138) *P*<**0.001**	1.042 (1.008-1.077) *P*=**0.015**
**Alcohol consumption, drinks/d**	1.345 (1.053-1.638) *P*<**0.001**	1.209 (1.142-1.279) *P*<**0.001**	1.204 (1.134-1.279) *P*<**0.001**
**PNPLA3 G-allele**	3.132 (1.783-4.482) *P*<**0.001**	1.988 (1.485-2.890) *P*<**0.001**	1.757 (1.251-2.468) *P*=**0.001**
**SERPINA1 Z-allele**	2.102 (-0.881-5.084) *P*=0.167	1.572 (0.855-2.890) *P*=0.145	1.822 (0.952-3.487) *P*=0.070

D	ACLD	CSPH
**Age, year**	1.056 (1.045-1.068) *P*<**0.001**	1.046 (1.033-1.059) *P*<**0.001**
**BMI, kg/m²**	1.105 (1.073-1.138) *P*<**0.001**	1.042 (1.008-1.077) *P*=**0.015**
**Alcohol consumption, drinks/d**	1.209 (1.143-1.280) *P*<**0.001**	1.204 (1.134-1.279) *P*<**0.001**
**No risk allele**	Reference	Reference
**One risk allele**	1.957 (1.454-2.634) *P*<**0.001**	1.733 (1.223-2.456) *P*=**0.002**
**Two risk alleles**	3.173 (1.344-7.491) *P*=**0.008**	3.407 (1.395-8.319) *P*=**0.007**

Supplementary Table-7. Multivariable linear and logistic regression analyses investigating the association of individual risk alleles with liver stiffness measurement (LSM), presence of advanced chronic liver disease (ACLD) and clinically significant portal hypertension (CSPH): (**A**) for PNPLA3 and covariables, (**B**) for SERPINA1 and covariables and (**C**) in a combined regression model for both PNPLA3 and SERPINA1. (**D**) Logistic regression model comparing patients with no risk allele (reference) to those with any and two risk alleles. Covariables were: Age (per year), BMI (per kg/m2), alcohol consumption (per drink per day).

Abbreviations:

ACLD advanced chronic liver disease
BMI body mass index
CSPH clinically significant portal hypertension
LSM liver stiffness measurement

Supplementary Table-8

A	LSM kPa	ACLD	CSPH
**Age, year**	0.087 (0.044-0.130)<br>*P*<**0.001**	1.048 (1.036-1.061)<br>*P*<**0.001**	1.036 (1.022-1.051)<br>*P*<**0.001**
**BMI, kg/m²**	0.273 (0.147-0.400)<br>*P*<**0.001**	1.111 (1.076-1.147)<br>*P*<**0.001**	1.041 (1.004-1.078)<br>*P*=**0.028**
**Alcohol abuse**	13.397 (11.773-15.022)<br>*P*<**0.001**	8.522 (5.976-12.153)<br>*P*<**0.001**	8.064 (5.565-11.686)<br>*P*<**0.001**
**Type 2 diabetes mellitus**	5.801 (3.790-7.813)<br>*P*<**0.001**	3.265 (2.162-4.931)<br>*P*<**0.001**	2.954 (1.875-4.655)<br>*P*<**0.001**
**PNPLA3 G-allele**	2.610 (1.353-3.867)<br>*P*<**0.001**	1.943 (1.420-2.659)<br>*P*<**0.001**	1.655 (1.154-2.373)<br>*P*=**0.006**

B	LSM kPa	ACLD	CSPH
**Age, year**	0.083 (0.040-0.127)<br>*P*<**0.001**	1.046 (1.034-1.059)<br>*P*<**0.001**	1.035 (1.021-1.049)<br>*P*<**0.001**
**BMI, kg/m²**	0.293 (0.166-0.421)<br>*P*<**0.001**	1.113 (1.079-1.150)<br>*P*<**0.001**	1.043 (1.007-1.081)<br>*P*=**0.018**
**Alcohol abuse**	13.634 (12.000-15.267)<br>*P*<**0.001**	8.683 (6.103-12.354)<br>*P*<**0.001**	8.463 (5.828-12.290)<br>*P*<**0.001**
**Type 2 diabetes mellitus**	5.913 (3.892-7.934)<br>*P*<**0.001**	3.332 (2.214-5.015)<br>*P*<**0.001**	3.028 (1.921-4.774)<br>*P*<**0.001**
**SERPINA1 Z-allele**	2.581 (-0.208-5.370)<br>*P*=0.070	1.791 (0.934-3.434)<br>*P*=0.079	2.181 (1.082-4.394)<br>*P*=**0.029**

C	LSM kPa	ACLD	CSPH
**Age, year**	0.086 (0.043-0.129)<br>*P*<**0.001**	1.048 (1.035-1.061)<br>*P*<**0.001**	1.036 (1.021-1.050)<br>*P*<**0.001**
**BMI, kg/m²**	0.279 (0.152-0.406)<br>*P*<**0.001**	1.112 (1.076-1.148)<br>*P*<**0.001**	1.041 (1.005-1.079)<br>*P*=**0.025**
**Alcohol abuse**	13.476 (11.851-15.101)<br>*P*<**0.001**	8.721(6.104-12.459)<br>*P*<**0.001**	8.391 (5.765-12.212)<br>*P*<**0.001**
**Type 2 diabetes mellitus**	5.801 (3.791-7.810)<br>*P*<**0.001**	3.283 (2.170-4.966)<br>*P*<**0.001**	2.982 (1.888-4.711)<br>*P*<**0.001**
**PNPLA3 G-allele**	2.618 (1.363-3.874)<br>*P*<**0.001**	1.960 (1.431-2.684)<br>*P*<**0.001**	1.678 (1.169-2.410)<br>*P*=**0.005**
**SERPINA1 Z-allele**	2.623 (-0.149-5.394)<br>*P*=0.064	1.865 (0.968-3.596)<br>*P*=0.063	2.255 (1.120-4.544)<br>*P*=**0.023**

D	ACLD	CSPH
**Age, year**	1.048 (1.035-1.061)<br>*P*<**0.001**	1.036 (1.022-1.050)<br>*P*<**0.001**
**BMI, kg/m²**	1.112 (1.077-1.148)<br>*P*<**0.001**	1.041 (1.005-1.079)<br>*P*=**0.025**
**Alcohol abuse**	8.735 (6.115-12.476)<br>*P*<**0.001**	8.385 (5.764-12.199)<br>*P*<**0.001**
**Type 2 diabetes mellitus**	3.285 (2.172-4.969)<br>*P*<**0.001**	2.971 (1.881-4.693)<br>*P*<**0.001**

**No risk allele**	Reference	Reference
**One risk allele**	1.941 (1.409-2.674) *P*<**0.001**	1.658 (1.145-2.402) *P*=**0.007**
**Two risk alleles**	3.791 (1.496-9.606) *P*=**0.005**	4.252 (1.620-11.162) *P*=**0.003**

Supplementary Table-8. Multivariable linear and logistic regression analyses investigating the association of individual risk alleles with liver stiffness measurement (LSM), presence of advanced chronic liver disease (ACLD) and clinically significant portal hypertension (CSPH): (**A**) for PNPLA3 and covariables, (**B**) for SERPINA1 and covariables and (**C**) in a combined multivariable regression model for both PNPLA3 and SERPINA1. (**D**) Multivariable logistic regression model comparing patients with no risk allele (reference) to those with any and two risk alleles. Covariables were: Age (per year), BMI (per kg/m2), active or past alcohol abuse (women ≥ 2 drinks per day, men ≥ 3 drinks per day), type 2 diabetes mellitus.

Abbreviations:

ACLD advanced chronic liver disease
BMI body mass index
CSPH clinically significant portal hypertension
LSM liver stiffness measurement
